# Partial Hepatectomy for the Resistant Fasciola Hepatica Infection in a Child

**Published:** 2015-09-01

**Authors:** Gülhan Belgin, Kanık Yüksek S, Tezer H, Özkaya Parlakay A, Dalgıç B, Dalgıç A, Yilmaz G

**Affiliations:** 1 Ankara Hematology Oncology Children's Training and Research Hospital, Pediatric Infectious Disease Unit, Ankara; 2 Gazi University School of Medicine, Pediatric Infectious Disease Unit, Ankara; 3 Gazi University School of Medicine, Gastroenterology and Hepatology Unit, Ankara; 4 Gazi University School of Medicine, General Surgery Unit, Ankara; 5 Gazi University School of Medicine, Pathology Unit, Ankara

**Keywords:** Fasciola hepatica, Children, Hepatectomy

## Abstract

Fascioliasis is an emerging and important chronic parasitic disease caused by two trematode liver fluke species: Fasciola hepatica (F. hepatica) and Fasciola gigantica (F. gigantica) infecting several herbivorous mammals including cattle, goats, sheep, and humans. We report a 9-year-old girl who suffered from F. hepatica infection and underwent right hepatectomy because of increasing abdominal pain resistant to anthelmintic chemotherapy. When anthelmintic drug treatment is not effective and abdominal pain persists, surgical resection including hepatectomy should be kept in mind for resistant F. hepatica infection.

## INTRODUCTION

Humans may ingest the infective larvae (metacercariae) by eating contaminated aquatic vegetables (especially watercress), by drinking contaminated water, or by washing vegetables and kitchen utensils with metacercariae-carrying water. After ingestion, the metacercariae pass into the peritoneal cavity, then enter the liver parenchyma (hepatic phase). They migrate through the liver parenchyma to enter the bile ducts, where they mature and release eggs (biliary phase). This process often lasts for 1 to 3 months.[1] Here we report a 9-year-old girl who suffered from F. hepatica infection and underwent right hepatectomy.

## CASE REPORT

A 9-year-old girl of Ankara, the capital of Turkey, presented with a complaint of abdominal pain, fever, and vomiting for one week. She had weight loss of about 2 kilograms in last two months. On examination right upper quadrant was mildly tender. A lesion in right lobe of liver was detected on ultrasound abdomen and she was hospitalized with a diagnosis of liver abscess. Complete blood count showed hemoglobin 11.6gm/dl, leukocyte count 11.3x109/L and platelet count of 385x109/L. Peripheral blood smear revealed 40% eosinophilia, 16% lymphocyte and 44% neutrophils. C-reactive protein was 10.3 mg/dl (normal range: 0- 0.8 mg/dl). Liver function tests showed aspartate aminotransferase 66 U/L (normal range: 15-60) and alanine aminotransferase 154 U/L (normal range: 15-46). Bilirubin levels, alkaline phosphatase, and gamma-glutamyl transferase were within normal limits. On ultrasound there was a linear hypoechogenic lesion sized 98 mm x 50 mm in right lobe of the liver in anterior part. There was also a multilobular hypodense 70 mm x50 mm x 78 mm sized lesion on segments 5, 6, and 7 on MRI. Serum immunoglobulin (Ig) E was slightly elevated.

Cephoperazone-sulbactam treatment was initiated for liver abscess. Serological analyses by ELISA and immunofluorescent antibody tests (IFAT) was negative for hydatid cyst. Indirect hemagglutination (IHA) revealed positivity for F. hepatica at a titration of 1/2560. Triclabendazole treatment was started at a dose of 10 mg/kg once a day and a second dose was given after ten days. Cephoperazone-sulbactam treatment was continued for 14 days and patient was discharged.

One month later, repeat USG revealed slight progression in size of the lesion (83 mm x 47 mm). She was again hospitalized because of abdominal pain and fever. Meropenem treatment (60 mg/kg/day) was started and given for one month. Triclabendazole treatment was given again at the same dosage. Liver trucut biopsy was performed for etiology. There was necrotic tissue and eosinophilic abscesses on biopsy. F. hepatica IHA decreased to 1/1280 after two months. She was discharged, but no resolution was observed again after one month follow-up. She needed multiple admissions for abdominal pain.

Surgical intervention was planned after consultation with surgery department and a right hepatectomy, including the segments 4, 5, 6, 7, and 8, was performed (Fig. 1). Microscopically, a cross-section of a partially degenerated F. hepatica was seen within the necrotic tunnel which was formed as the parasite travels through the liver parenchyma. The tunnel was composed of necrotic debris-like material, inflammatory cells, with many surface spicules of the parasite scattered around (Fig. 2). At follow-up, the patient recovered completely without any complaints or symptoms with IHA test negative for F. hepatica.

**Figure:**
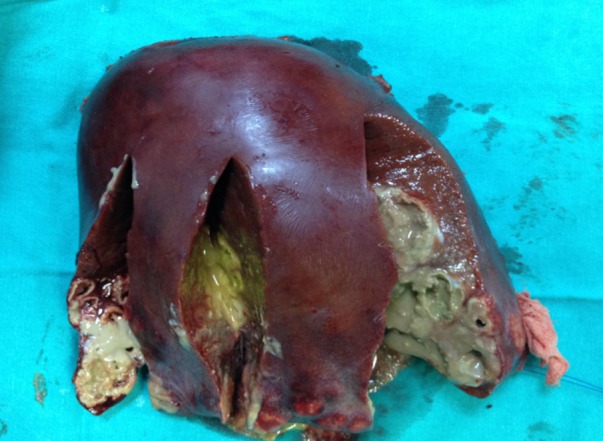
Figure 1:Right hepatectomy, including the segments 4, 5, 6, 7, and 8.

**Figure:**
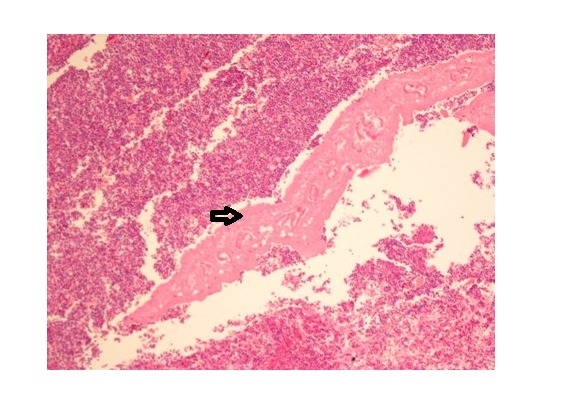
Figure 2:Cross-section of a partially degenerated F. hepatica was seen within the necrotic tunnel (arrow) which is formed as the parasite travels through the liver parenchyma. The tunnel was composed of necrotic debris-like material, inflammatory cells, and also many surface spicules of the parasite scattered around.

## DISCUSSION

Hepatectomy due to F. hepatica infection have been reported in adult patients. Yen et al reported a 36-year-old woman patient treated with right hepatectomy for a mass in liver. F. hepatica infection was found onhistopathology.[2] In the index case, the lesion in the liver was very large-sized but we suspected parasitic infection preoperatively. No resolution was achieved after anthelmintic drug treatment.

Definitive diagnosis of fascioliasis depends on serologic tests and/or demonstration of F. hepatica eggs in stool and/or bile samples. CT scan can depict two types, one consisting of an abscess-like lesion with single or multiple hypodense nodular areas, while the second consists of tunnel-like branching hypodense areas that are better delineated after contrast injection and is highly suggestive of fascioliasis in an appropriate clinical setting. Eosinophilia, positive IHA test for F. hepatica, and CT/US findings of our patient lead to the diagnosis of F. hepatica infection. Fascioliasis can be prevented with public education and environmental precautions such as avoiding consumption of contaminated water and plants. Prognosis is excellent with appropriate treatment.[3,4]

## Footnotes

**Source of Support:** Nil

**Conflict of Interest:** None declared

